# Using GPS and accelerometer data to precisely record egg laying, incubation and chick hatching of Cinereous Vultures (*Aegypiusmonachus*) in-situ

**DOI:** 10.3897/BDJ.13.e150787

**Published:** 2025-07-03

**Authors:** Cornel Cotorogea, Emilian Stoynov, Atanas Grozdanov, Simeon Marin, Hristo Valeriev Peshev, Ivelin Ivanov, Georgi P. Stoyanov

**Affiliations:** 1 Green Balkans, Stara Zagora, Bulgaria Green Balkans Stara Zagora Bulgaria; 2 Fund for Wild Flora & Fauna, Blagoevgrad, Bulgaria Fund for Wild Flora & Fauna Blagoevgrad Bulgaria; 3 Biology Faculty, Sofia University, Sofia, Bulgaria Biology Faculty, Sofia University Sofia Bulgaria; 4 Birds of Prey Protection Society, Sofia, Bulgaria Birds of Prey Protection Society Sofia Bulgaria

**Keywords:** vultures, incubation, nesting, chick rearing, GPS data

## Abstract

The Cinereous Vulture (*Aegypiusmonachus*) is a near-threatened species, making detailed monitoring of its breeding behaviour crucial for effective conservation. Traditional methods are often invasive and logistically challenging. This study presents a novel, non-invasive approach utilizing GPS and accelerometer data from tracking devices to precisely record key breeding parameters: egg-laying, incubation onset and duration, and chick hatching. This method allows for continuous, remote monitoring, minimizing disturbance to the breeding pairs.

The method was derived after a study on Cinereous Vultures in Bulgaria, using data from Ornitela GPS-GPRS transmitters (OT-30 and OT-50 models) deployed with leg-loop harnesses. Transmitters were configured to acquire data at intervals of 600 or 1200 seconds. Shorter intervals are recommended for optimal graph resolution. The methodology was developed based on observations of accelerometer graph patterns during the 2023 breeding season and validated through ground-truthing observations of GPS-tracked individuals (n=12, n=21 datasets) and laboratory experimental modelling and calculations in 2024. The study also retrospectively analyzed historical data, revealing previously undocumented hatching events and refining incubation timelines.

Accelerometer data derived from the tagged with GPS transmitters Cinereous Vultures, particularly along the X axis, provided distinct patterns corresponding to different breeding stages. During incubation, the X-axis values exhibited a specific, relatively broader range than the regular pattern, reflecting the bird's posture and limited movements while laying in the nest. During the incubation period, the birds are laid in the nest under a banking angle of +/- 10°-15°, alternating to the left and to the right. After hatching, the banking angle increases in the range +/- 15°-20°, when a significant increase in the X-axis range was observed, typically by a factor of 1.2 to 1.9, with most cases falling between 1.4 and 1.7 compared to the incubation period values. This increase reflects the parent's altered movements associated with chick care, such as warming, feeding, and nest maintenance. The intensive brooding and rearing period, lasting 16-23 days post-hatch, is characterized by the same high-amplitude X-axis pattern observed immediately after hatching. As the chick grows and requires less brooding, the accelerometer graph returns to pre-incubation values, indicating the adult's return to a more typical posture on the nest.

This methodology offers advantages over traditional monitoring techniques, providing continuous, precise, and non-invasive data acquisition. Its application can enhance conservation programs by enabling timely interventions, improving breeding success rates, and contributing to a deeper understanding of Cinereous Vulture reproductive biology. The potential for adapting this methodology to other vulture species, including Griffon and Bearded Vultures, warrants further investigation. Preliminary findings from limited 2024 data and historical data from Griffon Vultures suggest comparable accelerometer response patterns. This study demonstrates the power of combining GPS and accelerometer data to gain valuable insights into the breeding behavior of threatened avian species, ultimately aiding in their conservation.

## Introduction

The largest European bird of prey, tree nesting, and obligate scavenger, the Cinereous Vulture (*Aegypiusmonachus*) is a conservation-dependent species listed under the Near-threatened category on the Global level in the IUCN Red List (BirdLife International 2021). The reproductive biology of the species is well studied and known - pair formation and mating at the age of three years, first nesting at four years, nests built on tops of large trees, egg-laying in February-March, incubation of 51-57 days and fledging of the single chick in 110-140 days of hatching (Cramp and Simmons 1980). However, it is hard, if not impossible, to monitor the exact egg-laying date, hatching, and failures in the field, as the nests are in hardly accessible areas and sometimes out of direct sight. The new technologies, such as GPS transmitters, provide an invaluable insight into the bird's movements, whereabouts and behaviour (Peshev et al. 2021). The accelerometer data could also be used to detect a tracked bird's death (Kendall and Virani 2012, Sergio et al. 2018, Peshev et al. 2022), feeding and diet (Arkumarev et al. 2021a, Arkumarev et al. 2021b), nest site and incubation engagement (Ferraz et al. 2024), behavior and time-budget (Nathan et al. 2012, Sur et al. 2017, Patterson et al. 2019, García-Jiménez et al. 2020) e.g. setting up of on-line applications for automated notifications (Oppel et al. 2024, Ai2 2024).

Background Modern biologging technologies allow researchers to better understand animal movements, offering opportunities to measure survival and remotely study the breeding success of wild birds, i.e., by locating nests. This is particularly useful for species whose nests are difficult to find or access or when disturbances can impact the breeding outcome (Ferraz et al. 2024).

Since 2018, a reintroduction project has been ongoing in Bulgaria, whereby, in 2023, 71 Cinereous Vultures were released - all were equipped with GPS transmitters to follow their survival and whereabouts tightly (Ivanov et al. 2023). Tracking of the released Cinereous Vultures is an important tool to monitor them during the adaptation period, understand the territory use, foraging area, nest construction, migration pattern, threats and risks, pairing, etc. Close monitoring of the incubation periods in 2023 and 2024 revealed that important information can be obtained during the breeding period, with precise detection of incubation start and hatch.

Here, we report on findings of precise detection of egg-laying, hatching and failure in nesting of the Cinereous Vulture based on accelerometer data from the GPS transmitters. The method described and the information obtained would ease the monitoring of breeding pairs and provide clues for action to save the eggs/chicks or apply related interventions for the needs of that species conservation.

## Description

In the research on Cinereous Vulture reintroduction in Bulgaria, Ivanov et al. (2023) observed and ad-hoc described changes in the GPS-GPRS transmitters accelerometer graph patterns during the incubation period of two freshly formed and first-time-breeding pairs. This observation provided a clue for turning the accelerometer graph pattern change into an instrument for detailed remote detection of incubation appearance and its precise timing recording. The ad-hoc in-situ observed changes in the accelerometer values were then experimentally tested in a laboratory environment as described in detail in the Implementation section below. The results were compared with the in-situ obtained data from the transmitters, and intensive ground-truthing took place with field visits and contextual prolonged observations of the breeding performance of engaged and non-engaged in incubation GPS-tracked Cinereous Vultures (n=12 individuals, n=21 data sets - see Table 1).

**Figure 1.**
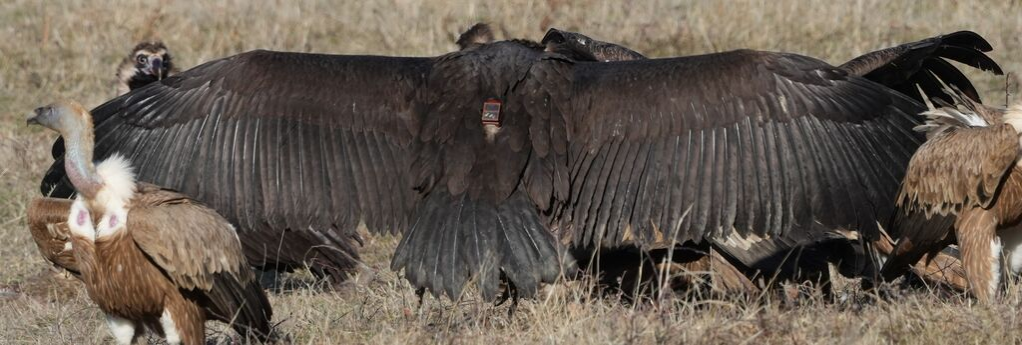
Cinereous and Griffon Vultures taking part in a feeding event in the wild. The GPS transmitter OT-50 mounted by a leg-loop harness is seen at the lower back of the Cinereous Vulture in the centre of the picture.

The birds in the study were equipped with Ornitela GPS-GPRS transmitters manufactured in 2019-2021, models OT-30 and OT-50 (2G and 3G versions) deployed with a leg-loop harness (Kenward 2004) in accordance to VCF's internal rules (Daniel Hegglin and Franziska Lörcher – pers. comm.) (Fig. 1).

The transmitters were set individually, depending on the model, age of the transmitter, battery and charge status. The typical interval for data acquisition of parameters was set to 600 seconds and, in some cases, to 1200 seconds. Longer intervals alter the graphs and are not recommended (in the case of low-battery trackers).

**Figure 2.**
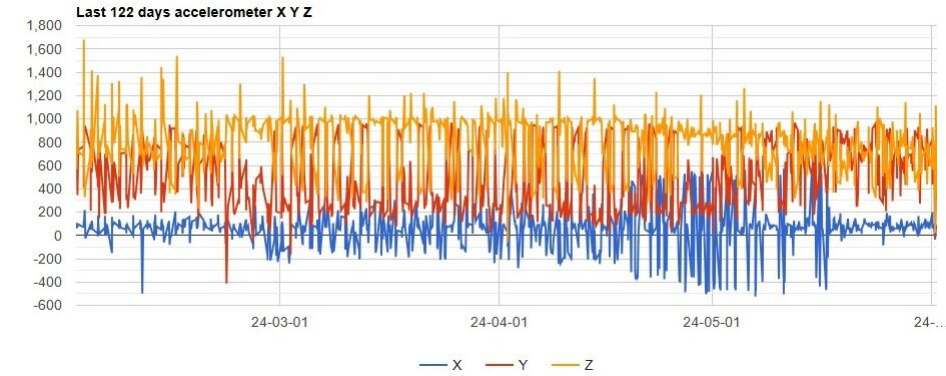
A four-month graph includes all the incubation and after-hatch periods for a successful breeding case. Three graph patterns are visible on the X axis (blue) from left to right: before incubation, which is a regular pattern; incubation - higher-amplitude blue bars; hatch and after - with further higher-amplitude blue bars, followed by a return to a regular pattern.

Close monitoring of the tracked breeding Cinereous Vultures during the 2023 breeding season revealed a significant pattern change observed during the breeding season on accelerometer data. Based on this observation, we started to analyze the changes in this pattern in detail, and we found that two typical graphs can be observed on the accelerometer X-axis values. The first period, consistent with the length of the incubation of 51-57 days, is followed by a visible change, which lasts for another 10-25 days, and then the graph pattern returns to regular. We concluded that this X-axis graph pattern changes (Fig. 2) are related to incubation, hatch and post-hatch period, and we started to analyze each case in detail with available data.

**Egg laying and incubation start**

The pairs were observed as the breeding season began, focusing on their behaviour. Attraction to a specific spot was a good indication of a potential nest. If nests were already built in the area, visual confirmation of their occupation and activity to upgrade was observed. Both birds, mainly males, were observed bringing construction material to the nest. About one month before breeding starts, copulations were observed.

If both birds were equipped with GPS transmitters before the female lays the egg, a steadier graph on accelerometer values could be observed, and the female would avoid making harsh moves. However, this change of pattern is hardly observed in all cases.

**Figure 3.**
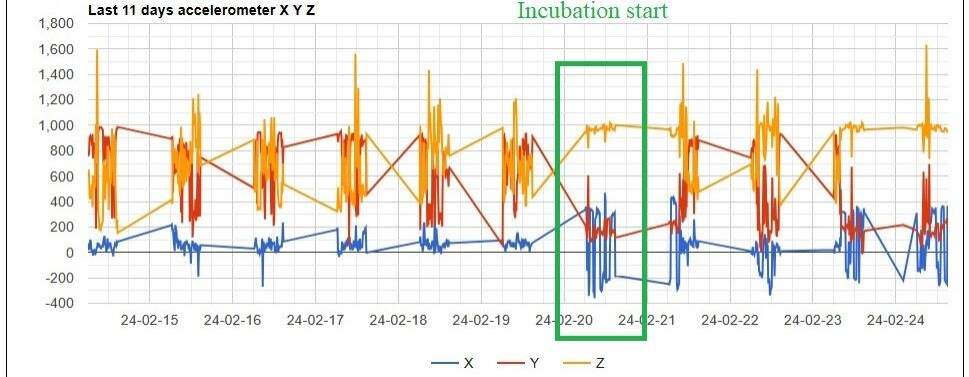
The incubation period started on 20.02.2024, and a significant X-axis pattern change—greater amplitude in the graph compared to the regular pattern—was observed.

After the egg is laid, incubation starts (Fig. 3). The first typical sign is the X-axis change; the graph shows positive and negative bars compared to the previous fluctuating median values. Soon, this bar graph pattern will become regular and can be observed on both - female and male birds as they alternate the incubation. During incubation, both the Y- and Z axes also show more steady characteristic values, with Z-axis values similar to typical values for flight mode (900-1000) caused by the near horizontal position of the tracker.

Monitoring of the incubation period was focused on the alternation of the birds, as the egg should be covered 100% for the entire period by one bird. One easy method is to put together both graphs of the male and female, but a more precise way is to analyze the daily tracks using Google Earth and CSV files (the time when the bird is positioned in the nest, based on GPS coordinates). Special care was provided for the new breeding pairs in the first week until they got into the incubation routine. Typical pair alternation on incubation is daily (sometimes even more times in one day), but longer shifts of 2-3 days of continuous incubation can be observed. When more extended incubation periods were observed, the bird not incubating was monitored by the GPS track online. Weather can play a role in alternation, sometimes preventing the pair from returning to the nest (snowstorm, fog, heavy rain).

**Hatching**

Based on the observation of the incubation start, hatching is expected to occur after 51-57 days (52-54 is typical). The hatch date is the next day after the first hatching signs arise.

**Figure 4.**
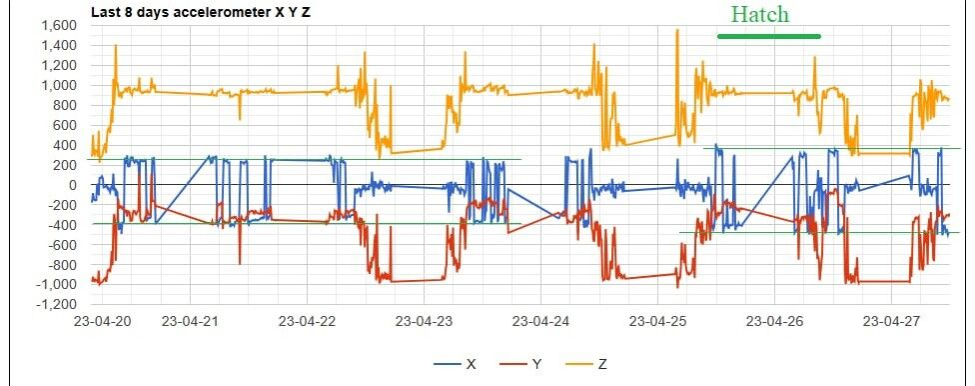
The chick hatched on 25.04.2023, with an increase in X-axis bars amplitude compared to the incubation period values.

The hatching is observed by a significant change in the accelerometer pattern, with the X-axis bars starting to have a higher amplitude (Fig. 4), 1.2-1.9 (1.5 average) times bigger than values during the incubation period. The amplitude change is a consequence of a shift in bird position, from direct contact to transfer body heat to the egg to a more distant position to allow the chick to move, but close enough to preserve the chick's body temperature as far as down feathers do not yet cover the chick.

Two days after hatch starts, when chick feeding begins, the graph pattern change becomes more pronounced. Care should be taken when concluding, as weather can affect the nest activity. During heavy rains, nest activity is reduced, and less movement is recorded as parents protect the chick. Similarly, in hot weather conditions, the bird in the nest might alternate positions and record more movement for cooling. However, the X-axis span remains at high values.

**Analysis of data from the accelerometer at the transition from incubation to hatched chick**

Even if the X-axis bar range change was noticeable, 21 data sets from incubation cases with confirmed successful hatch were analyzed. In each case, accelerometers behaved similarly, showing an increase in the range between the X-axis negative and positive peaks. The increase ranged from 1.2 to 1.9 times, with most cases in the range of 1.4 to 1.7 times.

Numerical data was extracted from CSV files saved from trackers, transmitting consistent data for 10 days around the hatching date. Data was filtered to show only peak X-axis values exceeding the thresholds, and average positive and negative peaks were calculated. The total span of the X-axis bars was calculated and used to calculate the ratio between the incubation period and after-hatching. The ratio between the after-hatching and incubation spans was always greater than 1, with estimated values in the interval 1.2—1.9. See Table 1.

**Figure 5.**
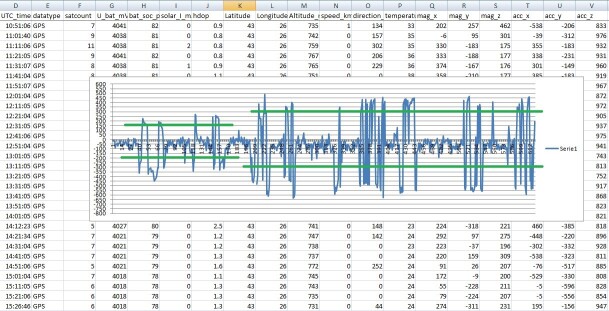
In this example, for the incubation period, values of -200 and +150 and values of -300 and +300 for the period after hatch were used as thresholds to filter the data (green lines on the graph). For each of the 21 cases, thresholds were adjusted according to each transmitter calibration.

Numerical data was extracted from CSV files saved from GPS transmitters, which transmitted consistent data for 10 days around the hatching date. The data was filtered to show only peak X values exceeding the thresholds, and the average positive and negative X-axis peaks were calculated. The total span of the X-axis bars was calculated and used to calculate the ratio between the incubation period and after hatching (Fig. 5).

The ratio between the after-hatching and incubation periods was always greater than 1, with calculated values in the interval 1.2—1.9, an average of 1.5 and most cases in the range 1.4 to 1.7 times (15 cases).

**Brooding and rearing period**

After the chick hatch is completed, for 16-23 days, brooding is observed by the same high amplitude X-axis pattern observed immediately after the hatch. After this period, as the chick grows and is completely covered and able to retain body temperature, the accelerometer graph of the adults starts to show typical values for a bird standing up on its feet (when in the nest). Then, the online observation of the nest shifted the focus from the graphs to the track. The presence of one adult in or near the nest was confirmed at least during the first two months after hatching.

## Implementation

To understand the bird's position and behaviour in the nest during the breeding period, an ex-situ and laboratory test was performed using an Ornitela OT-50 2GP transmitter to simulate identical values as observed on the tracker's accelerometer graphs from the breeding birds - specific for incubation and after hatch periods. Data available (graphs, CSV) was similar to data from the breeding vultures but with the advantage that tracker settings could be configured in advance, before making the tests. The settings' configuration used were identical to those used for regular monitoring of the vultures, except data session intervals, set to 3600 seconds. However, data session interval does not influence the parameters recorded but just the delay in time in which they are received by the OrniTrack control panel.

**Figure 6.**
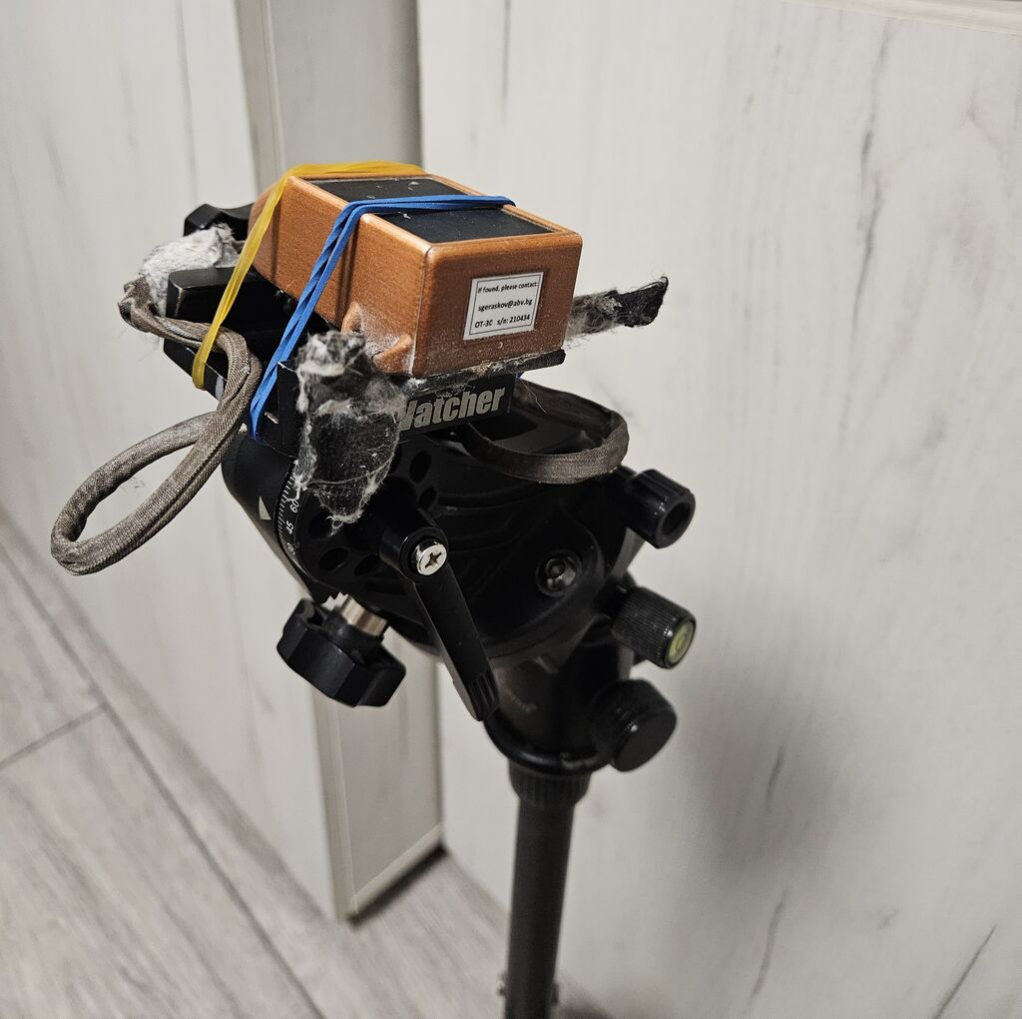
Ornitela OT-50 tracker used for tests in a laboratory.

The transmitter was installed on a camera tripod with a ball head and levelled for zero position and baseline for the graphs (Fig. 6). Tests were performed to determine the accelerometer X-axis response under random left and right banking angles to understand first the accelerometer response values to certain angles. Banking the transmitter to the left is considered a negative angle, producing a negative value on the X-axis, while banking to the right, regarded as a positive angle, creates a positive X-axis value. The interval between the lowest and highest X-axis values defines span. The span value is used to calculate the ratio of increase of X-axis bars from lower values corresponding to incubation to higher values corresponding to hatch and post-hatch period.

**Figure 7.**
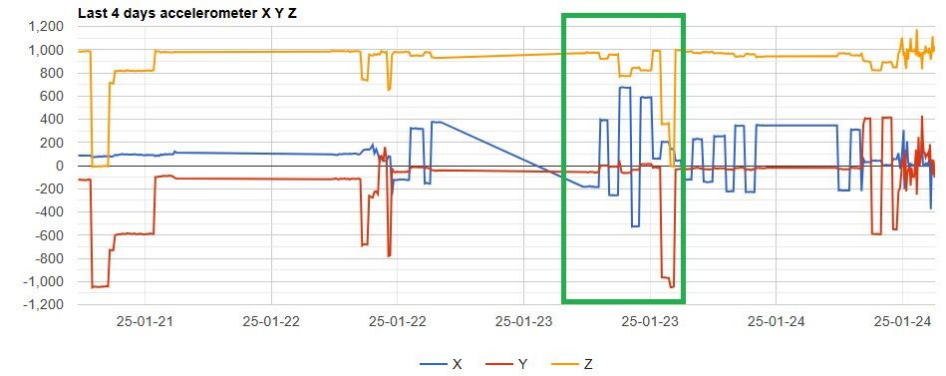
Tracker response when set on random angles for calibration.

For the simulation of X-axis values specific for the incubation period, approximate banking angles of +/-13° to +/-20° (measured after the angle was already set) were used for one hour (Fig. 7). The output X-axis graph replicates the X-axis bar graph observed during incubation. For the simulation of hatch and post hatch period, the bank angle was increased to almost double, ranging from +/-32° to +/-37° (approximate values).

### Methodology

**Experimental data**

The output X-axis graph showed a similar increase, proportional to the angle increase. Calculations were made, and the following results were obtained:

- Incubation

Total bank angle span=32°, X-axis span=574

- Hatching

Total bank angle span=68°, X-axis span=1196

Angle span ratio=2.12 produces X-axis span ratio hatch/incubation=2.08

**Figure 8.**
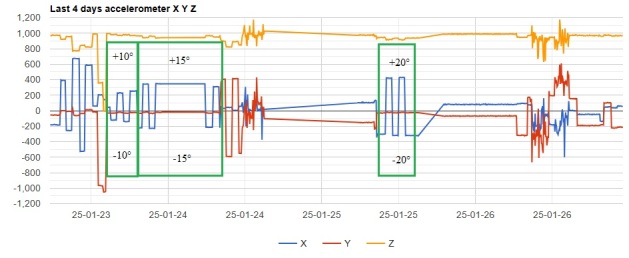
Tracker response to +/-10°, +/-15° and +/-20° bank angle.

These preliminary qualitative tests determined that an angle of +/-10-20° produces X-axis values in the range similar to those obtained from breeding bird trackers. After getting a good understanding of accelerometer response, a second stage of tests was performed at specified and more precise banking angles. The tracker settings were identical to the ones used to track the breeding birds in the wild, with a 600-second parameter acquisition interval. For each angle, +/-10°, +/-15° and +/-20°, a 4-hour test was performed, and all the data was downloaded in a spreadsheet file. The tracker angle was changed each hour, alternating left and right banking, corresponding to negative and positive angles, obtaining a bar graph identical to the ones observed on breeding bird trackers (Fig. 8).

Average values for X axis response were calculated for each banking angle, and based on the average values, span and ratio of increase were calculated. Data is summarized in Table 2.

- Incubation

Total bank angle span=20° (-10° to +10°), X-axis span=371

- Hatching

Total bank angle span=30° (-15° to +15°), X-axis span=570

Total bank angle span=40° (-20° to +20°), X-axis span=736

Angle span ratio=1.5 (10°-15°) produces X-axis span ratio hatch/incubation=1.54

Angle span ratio=2.0 (10°-20°) produces X-axis span ratio hatch/incubation=1.98

Based on the above data and data collected from the breeding pairs from season 2024 and earlier (Table 2), we conclude that during the incubation period, the birds are laid in the nest under a banking angle of +/- 10°-15°, alternating to the left and to the right. After hatching, the banking angle increases in the range +/- 15°-20°; see Fig. 9 as a reference from an online live camera.

Following these simulations, we conclude:

- During the breeding period, X-axis bars visible on the tracker's graphs result from tracker position, banked under a certain angle in the range of +/- 10°-20°. When banked to the left (considered a negative angle), the accelerometer X-axis values are negative, and when banked to the right (positive angle), the values of the X axis are positive;
- During the incubation period, the bank angle is generally in the range of +/- 10°-15° and, after hatching, is in the range of +/- 15°-20°;
- The X-axis span has a proportional evolution concerning banking angle. On average, a 1° deviation produces an 18-20 units deviation on the X axis;
- During hatching and after the chick was hatched, where real-life data showed an increase of X-axis bars and span by an average of ~1.5 times, it is proven now that a proportional increase of banking angle of the adult bird in the nest generates this increase;
- The data obtained while testing this tracker are relevant only for this tracker as absolute values, but the behaviour and trends apply to other similar models. Based on the testing procedure, we do not foresee a difference in the behaviour of the trackers deployed as leg loops compared with backpack deployment. There might be some differences in absolute values of the X, Y and Z axis, but the behavior should be similar.

### Application and Re-use potential

The methodology applied during the 2024 breeding season proved 100% reliable in cases where at least one breeding pair member carried a functioning transmitter capable of transmitting consistent data for analysis. All hatched chicks identified through this approach were visually confirmed. When hatching failed or did not occur, tracking data indicated the breeding pairs abandoned the nest post-failure.

Application to archived data revealed two additional hatched chicks that did not survive—one due to natural causes in 2022 and another from an unknown reason in 2021. Additionally, two incubation attempts were recorded in 2022 by the same breeding pair, with consecutive clutches spaced one month apart.

Further analysis of historical data enabled the precise determination of the incubation start and hatch dates for five fledged chicks from 2021 to 2023. In total, 14 hatched cases were documented, 12 of which were visually confirmed, and two were previously unknown at the time of occurrence.

In two cases, incubation and hatching were successfully monitored using data from a single transmitter, demonstrating the feasibility of this approach when only one bird carries a tracker. However, due to alternating nest duties, this method may have a one-day delay in detecting critical events, such as egg laying or hatching, when the untracked bird is in the nest.

The primary monitoring parameters are those provided by the 3-axis accelerometer sensor. Temperature readings can supplement accelerometer data to interpret bird behaviour, particularly during extreme weather events. For birds with problematic transmitters, relaxed settings should be used before the breeding season to maximize battery life. Once breeding begins, adjustments to 600- or 1200-second GPS fix intervals should be made. Monitoring over seven-day intervals is recommended, although other customized intervals may enhance pattern recognition.

This method was tested on different tracker models, with consistent recognition of behavioural patterns regardless of calibration variations. It has been validated for tracking egg laying, incubation, and hatching in Cinereous Vultures and retrospectively tested on previously confirmed hatched chicks using historical data. Tracking data and accelerometer readings also confirmed its effectiveness in determining unsuccessful breeding cases.

Preliminary findings suggest potential applicability to other vulture species. Limited 2024 data and historical data from Griffon Vultures (*Gypsfulvus*) equipped with similar trackers demonstrated comparable accelerometer response patterns. Further research on a larger dataset is required for validation.

Additionally, video evidence from breeding Bearded Vultures (*Gypaetusbarbatus*) suggests similar incubation behaviour, raising the possibility of extending this methodology to the species. If sufficient monitoring data is available, further analysis could confirm its feasibility. Assistance in data interpretation is available upon request.

The method could be adopted for inclusion in platforms for automatic detection, recording, and signalling for the breeding performance of tracked birds if added to software products such as EarthRanger and others.

## Data resources

Raw data used for this method development is presented as Excel files as Suppl. material 1.

Originally the data was retrieved as graphs with selectable time intervals, tracks in KML, KMZ, and GPX formats, and numerical data in CSV spreadsheet format. The retrieval of historical data is available as recorded, depending on each tracker configuration and settings at the moment of data transmission. In some cases, where available data was not sufficient for analysis due to the low battery of the trackers, forcing the use of longer data intervals for parameter acquisition, such data was not used to develop this method. In the case of trackers set to more than 1800-3600 seconds intervals between two sets of data, it is considered too long to observe the movement in the nest, both as accelerometer values and as track.

## Conclusions

The methodology developed using the accelerometer graphs' data to remotely identify incubation, hatching of chick and early stages of post-hatch chick rearing, discovered during the 2024 breeding season, has proven highly reliable for these breeding parameters monitoring in Cinereous Vultures. Experimental modelling and analysis of historical data have reinforced its accuracy and utility, uncovering previously unrecorded hatching events and improving precision in tracking incubation timelines. This approach may facilitate proactive monitoring and intervention, eventually enhancing breeding success rates.

Findings from unsuccessful breeding cases confirm the method’s ability to identify hatching failure patterns, including interrupted incubation, unsuccessful hatching, prolonged incubation without hatch indicators, and early chick mortality. These insights highlight environmental factors affecting breeding success, such as adverse weather conditions impeding adult foraging behaviour, disturbance, food shortages, etc.

This methodology could be reused for other vulture species, particularly Griffon and Bearded Vultures. It could also be applied to other large eagles, where preliminary data suggests similar behavioural patterns. However, further validation with larger datasets is required. The methodology is unsuitable for patagial trackers due to their wing-mounted positioning, which alters accelerometer readings.

Experimental validation using Ornitela OT-50 model trackers demonstrated the reproducibility of incubation and post-hatch behavioural patterns, confirming that accelerometer readings provide reliable insights into breeding behaviour. Data retrieval and monitoring through OrniTrack allow real-time and historical analysis, supporting adaptive conservation strategies.

Future application of this methodology can enhance conservation programs by providing precise incubation and hatching dates, enabling timely interventions for distressed birds. Its adaptability across tracker models and potential applicability to multiple vultures and large eagle species underscores its value as a tool for avian breeding studies and wildlife conservation.

## Supplementary Material

E998FEDF-E710-5481-BDFD-F50CF3221D98 10.3897/BDJ.13.e150787.suppl1 Supplementary material 1 Accelerometer data from GPS transmitters of 21 breeding events of 12 Cinereous Vultures Data type dates and accelerometer values Brief description The files provide raw data from the GPS transmitters of 21 data sets of 12 tagged Cinereous Vultures. File: oo_1253501.rar https://binary.pensoft.net/file/1253501 Cornel Cotorogea et al.

## Figures and Tables

**Figure 9a.**
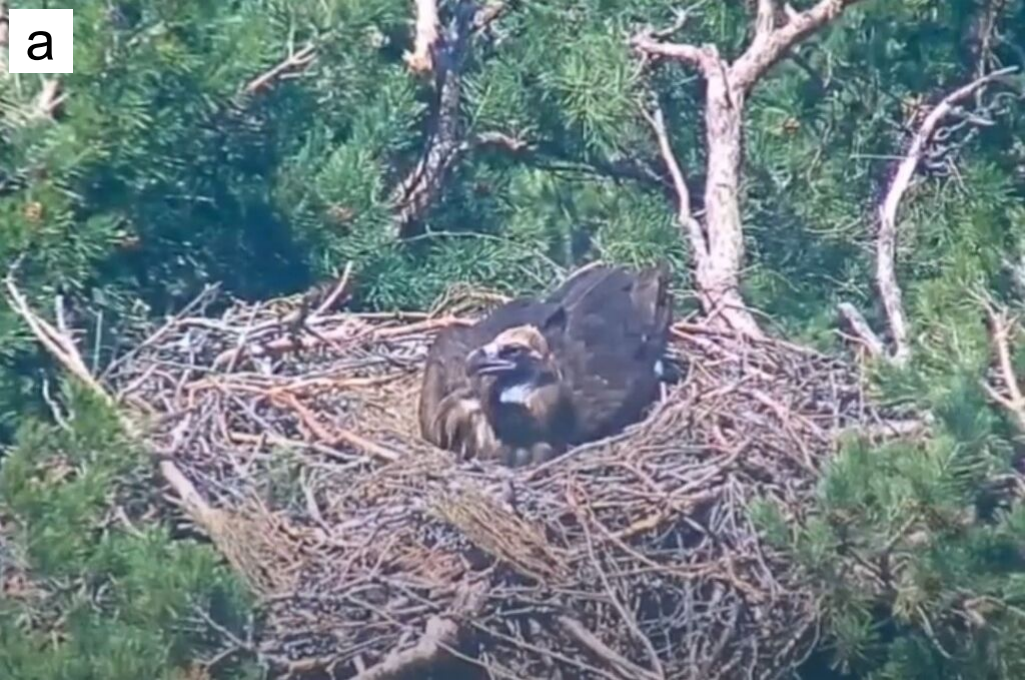
Incubating Cinereous Vulture in a nest - positioned banking to the left.

**Figure 9b.**
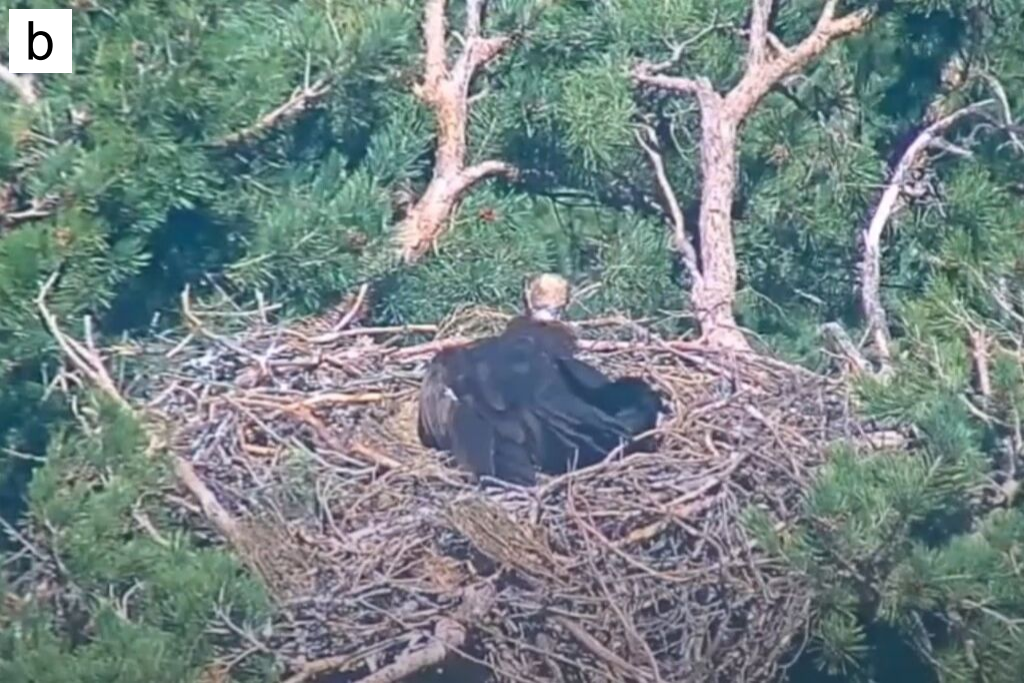
Incubating Cinereous Vulture in a nest - positioned banking to the right.

**Figure 9c.**
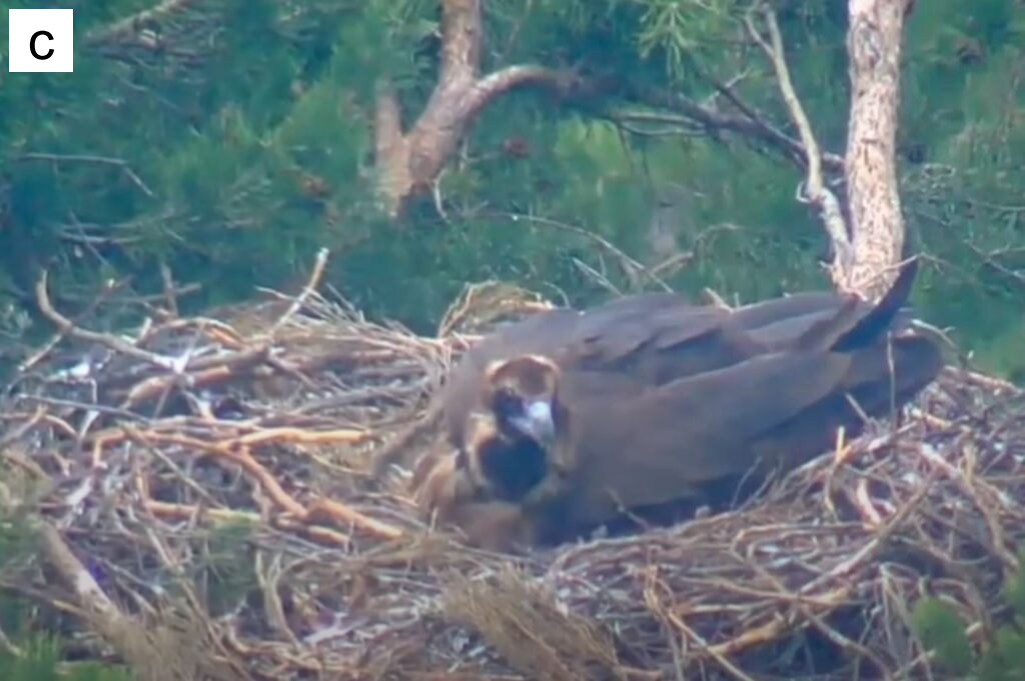
Another incubating Cinereous Vulture in a nest - posture banking to the left.

**Figure 9d.**
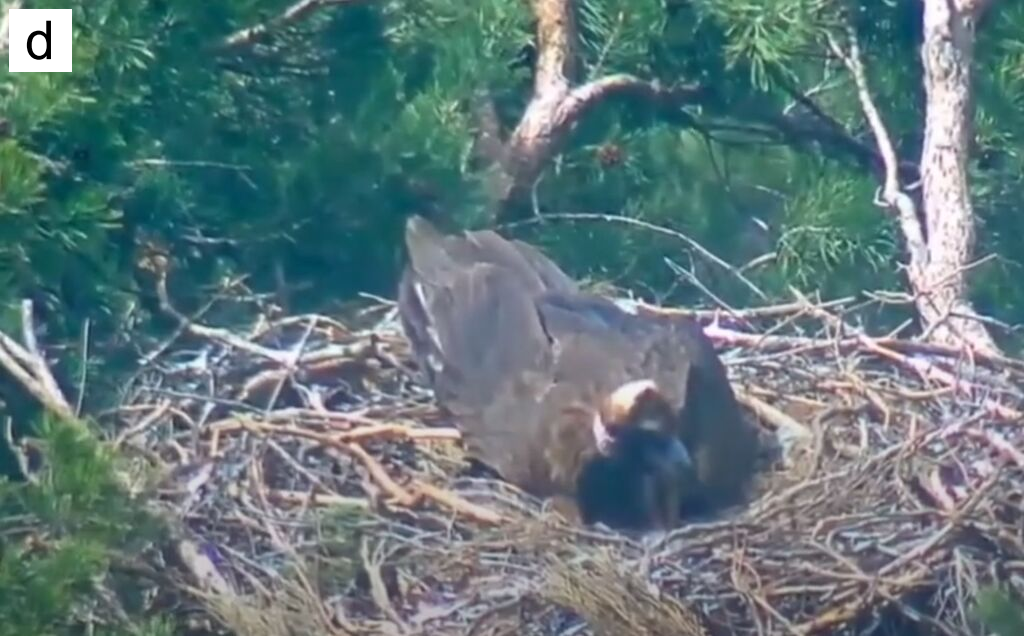
Another incubating Cinereous Vulture in a nest - posture banking to the right.

**Table 1.** Processed data sets from 21 transmitters from confirmed chick hatch cases during 2021-2024.

Name of the CV individual	Season	X axis						Ratio
		Before			After			
		Negative	Positive	Span	Negative	Positive	Span	
Balkan	2021	-243	269	512	-569	355	924	1.80
Kamchiya	2021	-326	283	609	-439	415	854	1.40
Vrachanski Balkan	2022	-204	303	507	-295	341	636	1.25
Kutelka	2022	-289	224	513	-400	319	719	1.40
Marto	2023	-263	389	652	-368	431	799	1.23
Kamchiya	2023	-315	224	539	-442	409	851	1.58
Kotel	2023	-386	295	681	-522	389	911	1.34
VCF Know-how	2023	-150	267	417	-361	432	793	1.90
Vrachanski Balkan	2023	-205	327	532	-325	461	786	1.48
Kutelka	2023	-384	270	654	-504	405	909	1.39
Kotel	2024	-323	213	536	-512	394	906	1.69
VCF Know-how	2024	-222	332	554	-347	483	830	1.50
Marto	2024	-278	364	642	-399	520	919	1.43
Balkan	2024	-322	273	595	-538	493	1031	1.73
Dobrudja	2024	-225	240	465	-398	412	810	1.74
Bagatur	2024	-308	210	518	-456	397	853	1.65
Karakachanka	2024	-227	324	551	-404	465	869	1.58
Vrachanski Balkan	2024	-236	390	626	-341	491	832	1.33
Kutelka	2024	-347	235	582	-468	374	842	1.45
Varshets	2024	-395	341	736	-472	407	879	1.19
Hristovich	2024	-329	314	643	-530	454	984	1.53
							Average	1.50

**Table 2.** Laboratory experimental data.

Angle	-10°	+10°	Span	-15°	+15°	Span	-20°	+20°	Span
X axis	-120	228	348	-222	346	568	-303	424	727
	-124	225	349	-222	343	565	-303	422	725
	-121	232	353	-223	341	564	-305	422	727
	-123	230	353	-223	343	566	-302	420	722
	-119	226	345	-219	345	564	-303	419	722
	-121	227	348	-221	341	562	-300	423	723
	-138	255	393	-229	349	578	-321	426	747
	-140	250	390	-228	350	578	-321	429	750
	-143	254	397	-227	346	573	-320	425	745
	-142	251	393	-225	347	572	-319	428	747
	-139	252	391	-229	348	577	-319	428	747
	-140	253	393	-228	344	572	-324	427	751
Average	-131	240	371	-225	345	570	-312	424	736
